# 

**Published:** 1874-11

**Authors:** 


### Contents of November Number.

### ORIGINAL DEPARTMENT.

ART. I.—Abstract of Proceedings of the Buffalo Medical Association, Oct. 6th, 1874. Reported by E. R. Barnes, M. D., Secretary...... 120
ART. II.—To what are the Changes in the Color of the Blood due. By E. M. Moore, M. D, ........................................ 126
ART. III.—Syphilitic Affections of the Nervous System. By Edward N. Brush, M. D., Continued,........................................ 130
ART. IV.—Clinical Remarks on Surgical Cases occurring at the Buffalo Hospital of the Sisters of Charity, by Prof. J. F. Miner, M. D. Reported by W. W. Miner, M. D. ........................................ 135
Correspondence, ........................................ 139

### MISCELLANEOUS.

The use of Ipecacuanha Spray in Winter Cough and Bronchitic Asthma,.... ........................................ 141
Some observations on the Local Action of Ipecacuanha,................ 147
Cincho-Quinine,.............. ........................................ 149
Discussion in regard to the use of Digitalis, Chloral and Bromide of Potassium,........................................ 151
The Condition of the Uterus five weeks after delivery, ................ 153
On Syphilitic Meningitis and Sypilitic Cerebral Disease,.......... .... 155

### EDITORIAL DEPARTMENT.

Obituary.—Dr. Daniel P. Bissell,........................................ 157
Books Reviewed :
The Building of a Brain. By E. H. Clarke, M. D.,.......... 158
Materia-Medica for the use of Students. By. John B. Biddle, M. D.,................. .................. .... 158
Conspectus of Medical Sciences. By E. H. Hartshorne, A. M., M. D.,........................................ 159
Archives of Dermatology. Edited by L. D. Bulkley, M. D.,. 159
Books and Pamphlets Received .... .. ............................ 160

THE BUFFALO

